# Transesophageal Echocardiography in Cardiogenic Embolic Cerebral Infarction

**DOI:** 10.12669/pjms.341.13291

**Published:** 2018

**Authors:** Wen Chu, Hua Wang

**Affiliations:** 1 Wen Chu, Ultrasonic Department, Luoyang Central Hospital Affiliated to Zhengzhou University, Henan, 471000, China; 2 Hua Wang, Ultrasonic Department, Luoyang Central Hospital Affiliated to Zhengzhou University, Henan, 471000, China

**Keywords:** Cardiogenic embolic cerebral infarction, Transesophageal echocardiography, Transthoracic echocardiography

## Abstract

**Objective:**

To evaluate the diagnostic values of transesophageal echocardiography (TEE) and transthoracic echocardiography (TTE) in cardiogenic embolic cerebral infarction.

**Methods:**

Fifty patients with occult cerebral infarction who were admitted to the hospital between June 2015 and June 2016 were selected as research subjects. The patients were diagnosed by transesophageal echocardiography and transthoracic echocardiography. Diagnostic data were compared to analyze the values of the two diagnostic methods.

**Results:**

Sixteen out of fifty patients were diagnosed as cardiogenic embolic cerebral infarction by TEE (32%), including two cases of aortic plaques, six cases of atrial septal defect, two cases of atrial septal aneurysm, two cases of patent foramen ovale, one case of left atrial spontaneous echo contrast, one case of mitral prolapse and two case of mitral stenosis. Four cases were diagnosed as cardiogenic embolic cerebral infarction by TTE (8.0%), including one case of patent foramen ovale, one case of left atrial spontaneous echo contrast, one case of mitral prolapse and one case of mitral stenosis. The difference was statistically significant (P<0.05). The main difference of TEE and TTE was detection of aorta atheromatous plaques and atrial septal lesions. Aortic atheromatous plaques of two cases and atrial septal lesions of eight cases were missed in the diagnosis by TTE.

**Conclusion:**

Detection and diagnosis of cardiac embolic cerebral infarction with TEE is highly accurate and advantageous. Therefore, TEE is worth promotion and application.

## INTRODUCTION

In recent years, patients with cerebral infarction are increasing year by year. As a result, it has been a common disease in clinics.^1^ Through investigation, Liu RF et al. found symptoms such as malacia and ischemic necrosis in the brain tissues of patients with cerebral infarction.^2^ If arteriosclerosis has appeared in cerebral tissues, blood circulation will be disordered and blood flow will slow down when the viscosity of blood abnormally increases. Once blood pressure decreases, fibrin and blood platelet in vascular tissues will deposit and be adhered to vascular walls to form thrombus, which leads to anoxia and ischemia in brain tissues and finally induces cerebral infarction.^3^,^4^ Cerebral infarction can be divided into different categories in clinics, and cardiogenic cerebral infarction is one of the most common cerebral infarction, with a high morbidity and mortality.^5^,^6^ A clinical study found that about 1/3 of cerebral infarction patients could not be definitely diagnosed.^7^ They were called occult cerebral infarction, and occult cerebral infarction was eventually diagnosed as cardiogenic cerebral infarction. Therefore, improving the diagnostic accuracy of cardiogenic cerebral infarction is of great clinical significance to the timely treatment.

A study suggested that echocardiography could effectively diagnose the pathogenesis of cardiogenic embolic cerebral infarction as well as the severity of disease conditions.^8^ Most cases of occult cerebral infarction are caused by heart problems. On account of this, this study investigated the practical values of transesophageal echocardiography and transthoracic echocardiography in the diagnosis of cardiogenic embolic cerebral infarction by diagnosing 50 patients with the two approaches.

## METHODS

Fifty patients with occult cerebral infarction who were admitted to the hospital between June 2015 and June 2016 were selected as the research subjects. All the patients satisfied the relevant diagnostic criteria of occult cerebral infarction and were confirmed as acute cerebral infarction by Magnetic Resonance Imaging (MRI). There were 29 males and 21 females; they aged from 22 to 56 years (average 38.3±3.4 years). Patients whose pathogenesis had been known (vasculitis, dissecting aneurysm, coagulation disorders and carotid artery stenosis > 50%) and who had definite heart diseases such as valvular disease, myocardial infarction, auricular fibrillation, congenital heart disease and arrhythmia were excluded. All the included patients signed informed consent. The study was approved by the ethics committee of the hospital.

All the patients underwent TTE and TEE. They received conventional electrocardiography and blood biochemical examination on admission, and moreover, the conventional data of the patients such as age, gender and stroke related risk factors were collected and recorded.

TEE was performed using Philip iE 33 ultrasonic apparatus whose frequency of single-plane TEE probe was 5.0 MHz. Firstly, patients who took left lateral position were anaesthetized with 1% lidocaine by surface spray twice. The breathing electrode and electrocardiograph displayer were connected. The probe was inserted following operation specification and then stopped in the middle of the esophagus and at the section of two atriums. The probe was rotated 180° to observe the anatomical structure and hemodynamic changes of the heart.

TTE was performed using Philip iE 33 ultrasonic apparatus whose frequency of probe was 5.0 MHz. The heart was examined through the four chamber view on the both sides of subcostal view. The main examination indicators included shunt speed and other deformity.

### Determination criteria

The severity of the disease was determined according to the examination results. Low-risk cardiogenic embolic cerebral infarction was determined if mitral stenosis, patent foramen ovale and atrial septal defect were observed, and high-risk cardiogenic embolic cerebral infarction was determined if severe left ventricular dysfunction, aortic atherosclerosis, left atrium tumor, severe mitral valve injury and left ventricular thrombus were observed.

### Statistical processing

Research data were statistically analyzed and processed using SPSS ver. 20.0. Enumeration data were expressed as percentage (%) and processed by Chi-square test. Difference was considered statistically significant if P<0.05.

## RESULTS

### Comparison of detection rates of cardiac embolic cerebral infarction

The detection rate of cardiac embolic cerebral infarction with TEE was much higher than that with TTE, and the difference had statistical significance (P<0.05). The difference of detection with TTE and TEE was reflected on aortic atheromatous plaques and atrial septum defect, as shown in Table-I.

**Table-I:** Comparison of detection rates of cardiac embolic cerebral infarction with TTE and TEE.

Item	TEE	TTE
Left atrial spontaneous echo contrast	1(2.0%)	1(2.0%)
Patent foramen ovale	2(4.0%)	1(2.0%)
Mitral stenosis	2(4.0%)	1(2.0%)
Mitral prolapse	1(2.0%)	1(2.0%)
Atrial septal aneurysm	2(4.0%)	0(0.0%)
Aortic plaque	2(4.0%)	0(0.0%)
Atrial septal defect	6(12.0%)*	0(0.0%)
Total	16(32.0%)*	4(8.0%)

* *Note:* indicated P<0.05 compared to TTE.

### Correlation between detection rate of cardiac embolic cerebral infarction and clinical characteristics

Among fifty patients with occult cerebral infarction, definite risk factors were not found in 11 patients, and 39 patients had at least one cerebral vessel associated risk factors. The detection rates of cardiac embolic cerebral infarction of patients with different genders and risk factors are shown in Table-II. The results suggested that cardiac embolic cerebral infarction was in correlation with hyperlipidaemia.

**Table-II:** Detection rate of cardiac embolic cerebral infarction and clinical characteristics.

	N	Number of cases which was detected out (%)
Female	21	7(33.3%)
Male	29	9(31.0%)
Smoking	14	4(28.6%)
Drinking	3	0(0.0%)
Hypertension	21	4(19.0%)
Diabetes	8	3(37.5%)
Hyperlipidaemia	17	11(64.7%)
Family history	8	2(25.0%)

### Effects of TEE results on treatment

The prevention and treatment of cardiac embolic cerebral infarction are special. The possible therapies for different types of cardiac embolic cerebral infarction concluded from literature are shown in Table-III. The patients immediately took 100 mg of aspirin after definite diagnosis of cerebral infarction. The treatment schemes of some patients were adjusted. Six patients who were found to have atrial septal defect were transferred to the department of cardiology of our hospital to undergo atrial septal defect occlusion and drug treatment, and none recurred during follow up. Two patients with aortic plaques were given enhanced statins treatment. Patients with left atrial spontaneous echo contrast, mitral prolapse and mitral stenosis turned to anticoagulation treatment following doctors' suggestions.

**Table-III:** TEE results and possible therapies.

TEE results	Possible therapy
Mitral prolapse	Treatment for arrhythmia; ultrasonic monitoring; anticoagulation
Mitral stenosis	Mitral valvuloplasty; anticoagulation
Left atrial spontaneous echo contrast	Oral administration of anticoagulation drugs
Patent foramen ovale	Aspirin; anticoagulation; surgery; interventional occlusion
Aortic plaques	Aspirin; statins
Atrial septal aneurysm	Anticoagulation; interventional occlusion
Atrial septum defects	Aspirin; anticoagulation; surgery; interventional occlusion

## DISCUSSION

Etiological diagnosis is an important step in the prevention and treatment of cerebral infarction. The current clinical data suggested that, about 1/3 of patients could be definitely diagnosed, and such cases were called occult cerebral infarction. With the constant advancement of medical technology level, abnormal heart function and structure are observed in some patients.^9^ Clinical data suggested that the effect of TEE was equivalent to that of transcranial doppler sonography in combination with TTE in the diagnosis of cardiogenic embolus,^10^ which though has not been certified through clinical examples but is enough to clarify the importance of diagnosis of cardiac function and structure abnormality with TEE.

TTE plays an important role in the diagnosis of cardiac cerebral infarction. However, TTE performs poorly in observing the structure of the heart close to spine, and moreover it can be affected by factors such as thickness of chest wall and obesity.^11^,^12^ Measuring the maximum diameter of the heart using TTE through four-chamber view and subcostal view is subject to be interfered by lung energy. Therefore, the application of TTE is greatly limited. TEE is a new technology for the diagnosis of cardiac cerebral infarction. Compared to TTE, TEE can detect cardiac cerebral infarction more accurately due to its specificity.^13^-^15^ Firstly, the position of patients and thickness of chest wall will not affect diagnosis with TEE; the diagnostic efficacy is excellent because TEE probe is close to the esophagus; moreover, it will not be affected by lung energy as the probe location is away from the lung. Secondly, TEE which is performed near the atrium can clearly observe the condition of the heart, and it is more sensitive in the diagnosis of cardiac cerebral infarction and plays an important role in the diagnosis of causes for cerebral infarction. In this study, the overall detection rate of occult cerebral infarction by TEE was 32.0%, higher than 8% by TTE, and moreover TEE was more sensitive in the detection of aortic plaques, patent foramen ovale, atrial septal aneurysm and atrial septal defect, indicating the high application values of TEE in the diagnosis of occult cerebral infarction.

The results of analysis on detection rates of cardiac embolic cerebral infarction and clinical characteristics suggested that hyperlipidaemia will produce significant impacts on the detection rate of cardiac embolic cerebral infarction, and the detection rate of cardiac embolic cerebral infarction among patients with hyperlipidaemia was 64.7%, indicating clinical characteristics could affect the detection rate of cardiac embolic cerebral infarction. To accurately determine the detection of cardiac embolic cerebral infarction with TEE and TTE, patients with clinical characteristics such as hyperlipidaemia should be excluded. It may be because air, liquid or solid embolus come in along with blood flow and block cerebral vessels, and hyperlipidaemia produces certain effect, which affects the detection of cardiac embolic cerebral infarction.

Cardiac ultrasonography can find possible cardiac embolic cerebral infarction. On account of it, more reasonable treatment method can be selected (Table-III). Two patients who were observed having aortic plaques were given enhanced treatment with anti-platelet aggregation and statins, six patients with atrial septal defect were transferred to the department of cardiology to undergo occlusion surgery, and the other eight patients were advised to undergo anticoagulation treatment. Effects of TEE results on treatment schemes are worth to be concerned.

## CONCLUSION

Detection and diagnosis of cardiac embolic cerebral infarction with TEE was highly accurate and advantageous, which can guarantee the timely treatment and improve the survival rate of patients. Therefore, TEE is worth promotion and application.

### Authors' Contribution

**WC & HW:** Study design, data collection and analysis.

**WC & HW:** Manuscript preparation, drafting and revising.

**HW:** Review and final approval of manuscript.

## References

[1] Qin YW, Teng T, He JQ, Du J, Tang CS, Qi YF (2013). Increased plasma levels of intermedin and brain natriuretic peptide associated with severity of coronary stenosis in acute coronary syndrome. Peptides.

[2] Liu RF, Luo YW, Gao F, Qi HY, An XL, Wang ZD (2015). Discussion on the correlation between the serum uric acid level of patients with acute cerebral infarction and short-time prognosis. Chin J Integr Med Cardio/Cerebrovas Dis.

[3] Mostofi K (2013). Neurosurgical management of massive cerebellar infarct outcome in 53 patients. Surg Neurol Int.

[4] Zhou X, Zhang C (2016). Clinical value of detection on serum monocyte chemotactant protein-1 and vascular endothelial cadherin levels in patients with acute cerebral infarction. J Acute Dis.

[5] Popovic D, Ostojic MC, Popovic B, Petrovic M, Vujisic-Tesic B, Kocijancic A (2013). Brain natriuretic peptide predicts forced vital capacity of the lungs, oxygen pulse and peak oxygen consumption in physiological condition. Peptides.

[6] El Zayat A (2014). Potential use of brain natriuretic peptide in patients with asymptomatic significant mitral stenosis. Egypt Heart J.

[7] Huang XQ, Jia JP, Fan CQ, Ma QF, Zhang Q (2013). Clinical and imaging characteristic of stroke patients with patent foramen ovale. J Neurosci Mental Health.

[8] Luo WQ, Huang ZY (2013). Application of transesophageal echocardiography in the diagnosis of patients with cardio-embolic cerebral infarction. Chin Mod Med.

[9] Kong FW, Kong Y, Li YZ, Kong FZ (2014). Imaging findings of cardiogenic cerebral embolism. Chin J Pract Nerv Dis.

[10] Wen Q, Liu RK, Yang CS (2015). The imaging characteristics, TOAST types and risk factors in youth cerebral infarction. Chin J Pract Nerv Dis.

[11] Wang HL, Xu TD, Li Y (2007). Transthoracic echocardiograph visualizing the efficiency of anticoagulant therapy for right atrium mobile thrombus in the elderly. Am J Emerg Med.

[12] Tang L, Li H, Niu M (2012). Application of transesophageal three-dimensional ultrasound and transthoracic echocardiography in the diagnosis of atrial septal defect. Friend Chem Indust.

[13] Mahon C, Keane D, Erwin J (2015). Incidence of thromboembolism following detection by trans-oesophageal echocardiography of left atrial thrombus. IJC Heart Vasculat.

[14] Champion S, Lenclud C, Deye N (2014). Pulmonary embolism related to central venous catheter triggered by trans-esophageal echocardiography bubble test: Caught red-handed!. Int J Cardiol.

[15] Yoshikawa H, Suzuki M, Hashimoto G, Kusunose Y, Otsuka T, Hara H (2013). Assessment of cyclic changes in the diameter of the aortic annulus using speckle-tracking trans-esophageal echocardiography. Ultrasound Med Biol.

